# Falls efficacy, postural balance, and risk for falls in older adults with falls-related emergency department visits: prospective cohort study

**DOI:** 10.1186/s12877-017-0682-2

**Published:** 2017-12-21

**Authors:** Yong-Hao Pua, Peck-Hoon Ong, Ross Allan Clark, David B. Matcher, Edwin Choon-Wyn Lim

**Affiliations:** 1 Department of Physiotherapy, Singapore General Hospital, Outram Road, Singapore, 169608 Singapore; 2 Research Health Institute, University of the Sunshine Coast, Sunshine Coast, Australia; 3 Health Services & Systems Research Program, Duke-NUS Graduate Medical School, Singapore, Singapore

**Keywords:** Balance, Falls efficacy, Falls

## Abstract

**Background:**

Risk for falls in older adults has been associated with falls efficacy (self-perceived confidence in performing daily physical activities) and postural balance, but available evidence is limited and mixed. We examined the interaction between falls efficacy and postural balance and its association with future falls. We also investigated the association between falls efficacy and gait decline.

**Methods:**

Falls efficacy, measured by the Modified Falls Efficacy Scale (MFES), and standing postural balance, measured using computerized posturography on a balance board, were obtained from 247 older adults with a falls-related emergency department visit. Six-month prospective fall rate and habitual gait speed at 6 months post baseline assessment were also measured.

**Results:**

In multivariable proportional odds analyses adjusted for potential confounders, falls efficacy modified the association between postural balance and fall risk (interaction *P* = 0.014): increasing falls efficacy accentuated the increased fall risk related to poor postural balance. Low baseline falls efficacy was strongly predictive of worse gait speed (0.11 m/s [0.06 to 0.16] slower gait speed per IQR decrease in MFES; *P* < 0.001).

**Conclusion:**

Older adults with high falls efficacy but poor postural balance were at greater risk for falls than those with low falls efficacy; however, low baseline falls efficacy was strongly associated with worse gait function at follow-up. Further research into these subgroups of older adults is warranted.

**Trial registration:**

ClinicalTrials.gov identifier: NCT01713543.

## Background

Falls are a common reason for older adults attending the emergency department (ED), and older adults with a falls-related ED visit have increased risk for subsequent falls, hospital readmission, functional decline, and mortality [1–5]. Accordingly, identifying the risk factors for falls in this clinical population is crucial [6].

Although low falls efficacy, defined as low self-perceived confidence in engaging in activities of daily living without falling, and impaired postural balance are putative risk factors for falls in community-dwelling older adults, the available data are limited and inconsistent [7–9]. We believe, however, that the mixed results of previous studies could reflect the complex interplay between falls efficacy, postural balance, and fall risk. Specifically, older adults with low falls efficacy may restrict their daily activities [9] – a strategy that may lower fall risk at least in the short term and diminish the association between postural balance and fall risk. However, this activity avoidance could potentially lead to functional decline over time. Conversely, while high falls efficacy is generally thought to be protective against falls, fall risk may be heightened among older adults with high falls efficacy but poor postural balance – a subgroup of “over-confident” older adults that has been largely neglected in previous falls studies [10–12].

Thus, our study aimed to examine the associations of falls efficacy, postural balance, and their interaction with fall risk (defined as the number of incident falls over a 6-month period) in a sample of older adults with falls-related ED visits. To further clarify the clinical significance of falls efficacy, we examined its association with future gait limitations.

## Methods

The current investigation was performed as a substudy of the **S**teps to **A**void **F**alls in **E**lderly (SAFE) trial (ClinicalTrials.gov NCT01713543) [13], a multicenter randomized trial that compared a home-based, customised programme versus the provision of an education booklet. Between December 2012 and June 2014, study personnel identified community-dwelling older adults (≥65 years) presenting to and discharged from the ED for a fall or fall-related injury. Exclusion criteria were an inability to follow the three-step command test, non-ambulatory status before the ED visit, and total blindness Trained research personnel travelled to participants’ home and assessed participants using a battery of self-report and functional measures at 3 and 9 months following hospital discharge. Due to limited personnel and funding, convenience sampling was used and the present study included 247 participants with available postural balance data at 3 months following hospital discharge (hereafter known as the baseline measure). Included participants were similar to those who were excluded because of missing postural balance data (data not shown). The study was approved by the institutional review boards of all participating sites, and all participants provided written informed consent.

### Falls efficacy and postural balance

We examined two primary independent risk factors: baseline falls efficacy and postural balance. Falls efficacy was measured with the 14-item Modified Falls Efficacy Scale (MFES) questionnaire [14]. Each item was scored on a 10-point Likert scale and the score of all answered items were summed and adjusted to a 0 to 140 scale, with higher scores indicating greater falls efficacy. The MFES was selected over the other scales because its items included daily outdoor activities on the use of public transport and road crossing. Standing postural balance was measured using computerized posturography on a Nintendo Wii Balance Board [15, 16], which has been shown to have concurrent validity with laboratory force plates [16]. Participants stood quietly on the balance board for 30 s and centre-of-pressure velocity along the antero-posterior (velocity-AP) and medio-lateral (velocity-ML) planes was recorded. Conceptually, centre-of-pressure velocity represents the amount of activity required to maintain stability during quiet standing; hence, greater velocity indicates poorer balance [17]. All participants performed 3 trials and the mean was taken.

### Covariates

To adjust for potential confounding (by risk factors for falls), we used subject-matter expertise and the literature [18–22] to a priori identify 6 covariates - namely, age [19], sex [18], fall history (yes/no) within the past 9 months (but excluding the most recent fall episode) [22], number of comorbidities [20], baseline Short Physical Performance Battery (SPPB) [21], and treatment group assignment. For the comorbidity measure, we used patient reports to aggregate the presence of 18 comorbid diseases such as hypertension, diabetes, congestive heart failure, coronary artery disease, stroke, Parkinson’s disease, and lung diseases. The SPPB [21, 23] is a measure of lower-extremity performance and comprises a tandem balance test, a timed 4-m gait speed test, and a timed chair stand test. Each test is scored from 0 to 4, and the total score ranges from 0 (worst performance) to 12 (best performance).

### Outcomes

The main outcome of the study was incident falls, defined as "an event which results in a person coming to rest inadvertently on the ground or floor of other lower level" [24]. Over a 6-month period, participants tracked monthly fall incidence on fall calendars [25, 26] and were contacted through monthly telephone calls [27]. In addition, we analyzed habitual gait speed, derived from the SPPB, at 6 months post baseline.

### Statistical analysis

Continuous variables were presented as means with standard deviation (SD)s and medians with interquartile range (IQR)s whilst categorical variables were presented as frequencies with percentages. The distributions of demographic and clinical factors in patients with and without a fall in the follow-up period were compared using the Wilcoxon rank-sum test or χ^2^ test, when appropriate.

The independent association of falls efficacy and postural balance with fall risk was assessed using a proportional odds regression model, with the number of falls as the dependent variable and MFES, center of pressure (CoP) AP-velocity, and CoP ML-velocity measures were each used as an independent variable. We adjusted all models for age, sex, fall history, number of comorbidities, baseline SPPB, and treatment group assignment. Proportional odds regression was chosen given its ability to handle zero-inflated falls data in this study [28, 29]. To evaluate the interaction between MFES and postural balance on the fall outcome, we tested for MFES × AP-velocity and MFES × ML-velocity interactions in separate models.

The independent association of falls efficacy with gait speed was assessed using a multivariable linear regression model, with gait speed as the dependent variable and MFES as the independent variable. The two postural balance measures served as covariates and to reduce collinearity, we used principal component analysis to combine them into one variable. Other covariates in the model were age, sex, fall history, number of comorbidities, baseline gait speed, and treatment group assignment.

For all analyses, to avoid assuming linearity, we modeled all continuous predictors as restricted cubic splines [28] unless there was insufficient evidence against the linearity assumption (*P* > 0.20). To minimize selection bias from missing predictor and outcome values, we performed predictive-mean-matching multiple imputation with 50 replications [28, 30]. In sensitivity analyses, we checked for interactions of treatment group assignment with falls efficacy and postural balance and found no statistically significant effect modification. We assessed the appropriateness of all models using residual plots, and we used *R* software, version 3.2.3 (http://www.r-project.org), for all analyses and graphing.

## Results

Table 1 shows the demographic and clinical characteristics of the sample. Of the 247 participants, 57 (24%) participants fell at least once, of whom 21 had two or more falls in the 6-month follow-up. Participants who fell at least once were more likely to report having a previous fall, have lower baseline SPPB scores, and walk more slowly at 6 months.

**Table 1:** Demographics and clinical characteristics

	Non-fallers (*n* = 190)	Fallers (*n* = 57)	Overall (*n* = 247)	*P*-value
Age (years)	71.0 **77.0** 83.0 (77.3 ± 7.3)	74.0 **78.0** 83.0 (78.2 ± 6.2)	73.0 **77.0** 83.0 (77.5 ± 7.1)	0.38^1^
Women	75% (143)	74% (42)	75% (185)	0.81^2^
Stroke or Parkinson’s disease	6% (12)	9% (5)	7% (17)	0.53^2^
Number of comorbidities	1.0 **2.0** 4.0 (2.6 ± 1.6)	2.0 **3.0** 4.0 (2.8 ± 1.6)	1.0 **2.0** 4.0 (2.6 ± 1.6)	0.32^1^
History of previous falls	43% (81)	60% (34)	47% (115)	0.024^2^
Baseline SPPB^a^	4.0 **7.0** 9.0 (6.8 ± 3.1)	3.0 **5.0** 8.0 (5.7 ± 3.0)	4.0 **7.0** 9.0 (6.5 ± 3.1)	0.025^1^
Baseline MFES^b^	78,**115**,128 (100 ± 34)	66,**104**,128 (95 ± 33)	76,**112**,128 (99 ± 34)	0.35^1^
Baseline CoP^c^ Velocity-AP^d^ (cm/s)	0.73 **0.91** 1.24 (1.11 ± 0.66)	0.75 **1.03** 1.44 (1.26 ± 0.81)	0.73 **0.95** 1.29 (1.15 ± 0.70)	0.23^1^
Baseline CoP^c^ Velocity-ML^e^ (cm/s)	0.45 **0.54** 0.69 (0.64 ± 0.37)	0.49 **0.58** 0.85 (0.70 ± 0.31)	0.45 **0.54** 0.74 (0.65 ± 0.35)	0.066^1^
Gait speed at 6 months (m/s)	0.42 **0.61** 0.81 (0.59 ± 0.26)	0.31 **0.50** 0.64 (0.50 ± 0.25)	0.38 **0.56** 0.77 (0.57 ± 0.26)	0.008^1^

Continuous variables are summarized as 25th 50th 75th percentiles and mean ± SD

Median value is captured in boldface

^1^Wilcoxon rank sum test; ^2^Pearson’s χ^2^ test

^a^SPPB – short Physical Performance Battery; ranges from 0 to 12, with higher scores indicating greater physical functioning

^b^MFES – modified Falls Efficacy Scale; ranges from 0 to 140, with higher scores indicating greater falls efficacy

^c^CoP – centre of pressure

^d^AP – anteroposterior

^e^ML – mediolateral

**Fig. 1:**
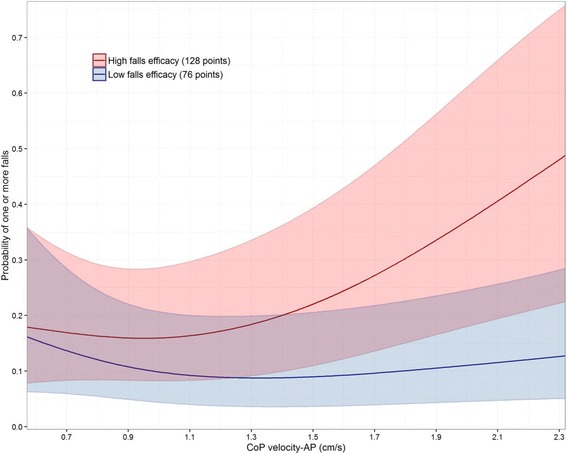
Interaction plot of Modified Falls Efficacy Scale (MFES) and centre-of-pressure (CoP) velocity-AP and probability of falling at least once in the follow-up period. Low and high falls efficacy represent the 25th and 75th percentile values of the MFES, respectively. Predicted fall risk was computed from a proportional odds model which included the interaction between MFES and CoP velocity-AP, adjusted for age, sex, number of comorbidities, fall history, baseline SPPB, and treatment group assignment (*P* = 0.014 for interaction). Shaded regions represent 95%CIs for the natural spline-smoothed estimates

When adjusted for covariates, there was no significant association between fall risk and MFES (interquartile range-odds ratio [IQR-OR], 1.58 [CI, 0.79 to 3.12], *P* = 0.19), velocity-ML (IQR-OR, 1.00 [CI, 0.77 to 1.29]; *P* = 0.98) or velocity-AP measures (IQR-OR, 1.05 [CI, 0.82 to 1.36]; *P* = 0.69). The MFES × velocity-ML interaction was of borderline significance (*P* = 0.076) while MFES interacted significantly with velocity-AP measure to influence fall risk (*P* = 0.014). Specifically, when MFES level was high, greater CoP velocity-AP was closely associated with greater fall risk; however, this association weakened with decreasing MFES levels (Fig. 1).

**Fig. 2:**
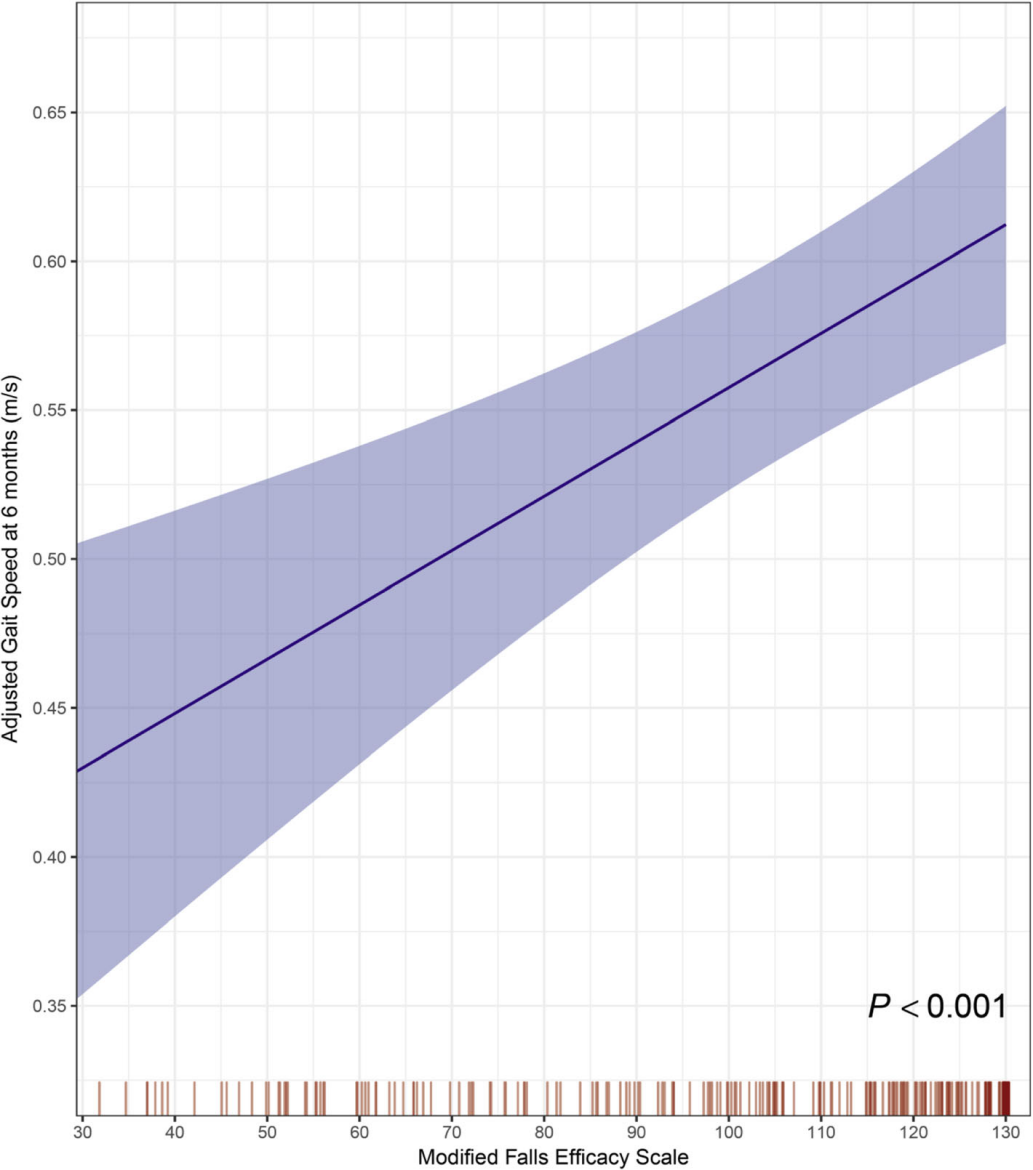
Lower scores on the Modified Falls Efficacy Scale (MFES) were independently associated with slower (worse) gait speed at 6 months post-baseline assessment, after adjustment for age, sex, number of comorbidities, fall history, baseline gait speed, treatment group assignment, and postural balance. Shaded regions represent 95% CI for the point estimates. Rug plots show the observed baseline MFES values

We then tested whether baseline falls efficacy was associated with gait speed at 6 months post baseline. After adjustment for covariates, lower MFES was an independent risk factor for future gait limitations (0.11 m/s [0.06 to 0.16] slower gait speed per IQR decrease in MFES; *P* < 0.001) (Fig. 2).

## Discussion

In this prospective study of 247 older adults, baseline falls efficacy interacted with postural balance to influence fall risk. These findings could shed light on the limited and mixed evidence regarding the association of falls efficacy and postural balance with fall risk: if low falls efficacy blunted the association between postural balance and fall risk, the “averaged effects” of falls efficacy and postural balance on fall risk could be biased toward the null.

Reviewing the literature, although falls efficacy has been reported to be significantly reduced in fallers compared to non-fallers [22, 31–33], it was not significantly lower amongst fallers in our study (Table 1). However, we have found that low baseline falls efficacy was strongly predictive of worse gait function (Fig. 2). To explain this phenomenon, it is possible that older adults with low falls efficacy may restrict their daily activities [9] to reduce their fall risk and are less likely to engage in exercises [34] – making them prone to postural instability and muscular weakness associated with deconditioning [35]., Potentially, this sequela of activity restriction may lead to increased long-term fall risk [35, 36]. Conversely, another interpretation is that a slow gait reflects a cautious gait strategy [37] and recent evidence also suggests that the generally low falls efficacy in Chinese older adults is protective against falls [38]. We believe, however, that this alternative explanation is less likely because our participants who fell in the follow-up period had relatively lower baseline SPPB scores and slower follow-up gait speed (Table 1). Nevertheless, to better clarify the causal pathway(s) of our findings, future work should examine the long-term fall risk in older adults with low falls efficacy.

Our results suggest that fall risk was heightened in older adults with high falls efficacy but poor postural balance. This finding is biologically plausible because “over-confident” older adults are also likely to walk fast (Fig. 2) and less likely to restrict their daily activities, and these factors may interact to increase fall risk [39, 40]. Although Delbaere and colleagues [34] found that their “under-fearful” older adults had similar, if not lower, fall risk compared with older adults who had an accurate perception of their high fall risk, we should emphasize that all our participants had a fall that led to an ED visit. Hence, we speculate that the combination of poor postural balance and high falls efficacy after a recent (~3 months) traumatic fall may more closely reflect a risk taking behaviour or an inaccurate fall-risk appraisal. Nevertheless, we acknowledge that a deeper investigation of our findings would require additional information on physical activity levels and risk-taking behaviour [12], and future work should explore this.

Our study has limitations other than those described earlier. First, although we studied a clinically relevant group of high fall-risk Asian older adults [6], it is unclear whether our results would persist in (i) a non-Asian sample or (ii) a broader sample that includes healthy older adults with no fall history at baseline. Second, whilst we had details about injurious falls and obtained qualitatively similar results (data not shown), our analyses were not sufficiently powered to draw firm conclusions.

## Conclusions

In summary, we observed that older adults with high falls efficacy but poor postural balance were at greater risk for falls than those with low falls efficacy; however, low falls efficacy was strongly predictive of future gait limitations. Further research into these subgroups of older adults is warranted to uncover ways in which their risk factors may be modified.

## Abbreviations

ED: Emergency department
MFES: Modified Falls Efficacy Scale
SAFE trial: **S**teps to **A**void **F**alls in **E**lderly trial
SPPB: Short Physical Performance Battery
velocity-AP: Velocity along the antero-posterior plane
velocity-ML: velocity along the medio-lateral plane
